# Does the Instability of Pertrochanteric Fractures in the Elderly Correlate With Weakened Gluteal Muscles?

**DOI:** 10.7759/cureus.72159

**Published:** 2024-10-22

**Authors:** Mitsuaki Noda, Shunsuke Takahara, Shinya Hayashi, Atsuyuki Inui, Keisuke Oe, Shin Osawa, Takehiko Matsushita

**Affiliations:** 1 Department of Orthopaedics, Himeji Central Hospital, Himeji, JPN; 2 Department of Orthopaedics, Hyogo Prefectural Kakogawa Medical Center, Kakogawa, JPN; 3 Department of Orthopaedic Surgery, Kobe University Graduate School of Medicine, Kobe, JPN

**Keywords:** ao/ota classification, computed tomography (ct), density, fracture type, gluteal muscle, gluteus maximus, muscle cross-sectional area, pertrochanteric fracture

## Abstract

Introduction

Suboptimal outcomes were observed in cases of unstable pertrochanteric fractures, even when bone healing occurs without complications. This raises the question of whether factors beyond bone health integrity, such as the frailty of muscles, contribute to these less favorable results. Muscles, particularly the gluteal muscles, not only influence functional ability but also serve as a cushion that provides physical protection against external forces during falls. When gluteal muscles are thin or weak, their ability to absorb the impact from a fall diminishes, potentially leading to unstable fractures. In this study, we compared gluteal muscle-related indices, including cross-sectional area (CSA) and muscle density, between stable and unstable pertrochanteric fractures. The aim of this study was to employ a retrospective approach to investigate the causes of unstable fractures, with a focus on potential muscular pathology. It was hypothesized that reduced CSA and lower density of the gluteal muscles would be associated with unstable fractures.

Material and methods

Geriatric patients aged 70 years or older with pertrochanteric fractures requiring surgical intervention were retrospectively identified from databases. These fractures classified as stable (A1) or unstable (A2) based on the Revised Arbeitsgemeinschaft für Osteosynthesefragen (AO)/Orthopaedic Trauma Association (OTA) Classification were compared based on demographic variables (age, height, body mass index (BMI), and fracture laterality) as well as muscle-related indices. A multivariate logistic regression model was employed to identify predictors of unstable fractures. Independent variables included age at the time of surgery, height, BMI, and muscular metrics CSA and density of the gluteus maximus and gluteus medius.

Results

Out of 220 patients identified from the database, 111 patients with an average age of 88 years (ranging from 71 to 103 years) were included. This cohort consisted of 40 patients with stable fractures (A1) and 71 patients with unstable fractures (A2). Among the demographical data, only fracture laterality demonstrated a significant difference between the groups (p < 0.05). Regarding gluteal muscle data, the CSA/BMI of both the gluteus maximus and medius, as well as the density of the gluteus medius, did not show significant differences between the two groups. The only exception was the density of the gluteus maximus, which was significantly lower in the unstable group (p < 0.01). A binary logistic regression analysis was conducted to identify risk factors for unstable fractures. The analysis found that the density of the gluteus maximus was a statistically significant predictor, with an odds ratio of 0.959 (95% CI, 0.923 to 0.997; p = 0.03). To determine an optimal cut-off value, receiver operating characteristic (ROC) analysis was performed for the density of the gluteus maximus. The Youden index identified a cut-off value of 20.8 HU for the gluteus maximus density as the optimal threshold (area under the curve (AUC): 0.625; 95% CI: 0.520-0.730).

Conclusion

This retrospective study investigated whether unstable pertrochanteric fractures in elderly female patients were linked to weakened gluteal muscles, compared to stable fractures, and suggested this muscle weakness may contribute to poor functional outcomes. Our binary regression analysis indicated that decreased muscle density in the gluteus maximus increases the risk of unstable fractures.

## Introduction

Pertrochanteric fractures are among the most common surgically treated injuries in the elderly population, with global incidences projected to reach 6 million by 2050 [1]. These fractures frequently lead to high rates of morbidity, mortality, and disability. Poor postoperative outcomes are particularly evident in unstable fractures, which could be assessed through functional measures like the Parker Mobility Score and the Barthel Index [2,3]. Various fixation techniques have been introduced to improve outcomes; however, even with optimal fixation, results are often suboptimal [4]. For instance, while fixation of the lesser trochanter may reduce complications like ischiofemoral impingement, it does not guarantee favorable outcomes in all cases [5].

Considering the suboptimal outcomes observed in cases of unstable fractures, even when bone healing occurs without complications, this raises the question of whether factors beyond bone health integrity, such as the frailty of soft tissues, including muscles, contribute these less favorable results. Despite the known impact of muscle on functional recovery, little research has examined the differences in gluteal muscle condition between stable and unstable pertrochanteric fractures in elderly patients [6,7]. Muscles, particularly the gluteal muscles, not only influence functional ability but also serve as a protective cushion against external forces during falls, which are common among older adults [8,9]. When gluteal muscles are thin or weak, their ability to absorb the impact from a fall diminishes, potentially leading to more complex and unstable fractures [8].

In this study, we compared gluteal muscle-related indices, including cross-sectional area (CSA) and muscle density, between stable and unstable pertrochanteric fractures. The underlying causes of unstable fractures remain poorly understood. The aim of this study was to employ a retrospective approach to investigate the causes of unstable fractures, with a focus exclusively on potential muscle within soft tissue. It was hypothesized that reduced CSA and lower density of the gluteal muscles would be associated with unstable fractures. If this assumption holds true, the poorer postoperative outcomes observed in unstable fractures may be attributed to muscular weakness.

## Materials and methods

Study design

Geriatric patients aged 70 years or older with pertrochanteric fractures requiring surgical intervention, treated between January 2013 and January 2024, were retrospectively identified from trauma databases at Nishi Hospital, Kobe, and Himeji Central Hospital, Himeji, Japan. To be included in the study, patients needed complete data sets, including preoperative two-view radiographs (anteroposterior and lateral) and 3D computed tomography (CT) scans, along with demographic information. The study focused on fractures resulting from low-energy trauma, such as falls from standing height. Exclusion criteria were as follows: (1) patients younger than 70 years at the time of surgery, (2) fractures classified as type A3 according to the Revised Arbeitsgemeinschaft für Osteosynthesefragen (AO)/Orthopaedic Trauma Association (OTA) Classification [10], (3) history of surgery on the contralateral hip, (4) male patients (due to insufficient sample size for meaningful analysis), (5) patients treated conservatively, (6) fractures from high-energy trauma such as traffic accidents or falls from heights, and (7) major nerve or spinal cord conditions that could significantly impact physical performance.

The study was approved by the Ethics Committees of both hospitals: Nishi Hospital Ethics Committee (approval number 2021-1) and Himeji Central Hospital Ethics Committee (approval number 2024-43).

Outcomes and assessments

Fractures were classified as stable (A1) or unstable (A2) based on the Revised AO/OTA Classification for pertrochanteric fractures, evaluated by an experienced surgeon using both CT scans and plain radiographs. Two groups, stable and unstable fractures, were compared based on demographic variables (age, height, body mass index (BMI), and fracture laterality) as well as muscle-related indices. BMI was calculated as weight divided by height squared and was also used to adjust for muscle area in each patient’s physique.

A multivariate logistic regression model was employed to identify predictors of unstable fractures. Independent variables included age at the time of surgery, height, BMI, and muscular metrics CSA and density of the gluteus maximus and gluteus medius, chosen for their clinical relevance [11]. Data on comorbidities, medications, and serum chemistry were not collected for this study. The authors periodically reviewed and discussed the data and manuscript, either in person or online, to finalize the content.

CT assessment of muscle indices

The two hospitals used different CT acquisition parameters. At Nishi Hospital, preoperative CT scans were obtained from the iliac crest to just below the lesser trochanter (120 kVp, Auto mA, Noise Index 9.5), with 5.0-mm-thick slices at 5.5-mm intervals. The Optima CT 660 CT Scanner (GE Healthcare, Tokyo, Japan) was used, and images were processed with AZE Virtual Place Fujin Raijin software (Version 3.0, AZE, Tokyo, Japan). At Himeji Central Hospital, CT scans were performed under similar conditions (120 kVp, Auto mA, Noise Index 8.0) with 5.0-mm-thick slices at 5.0-mm intervals. The Aquilion Prime Multi-Slice System (Canon Medical Systems, Tochigi, Japan) was utilized, and images were analyzed using Ziostation Revoras software (Version 5.2.0.0, Ziosoft, Tokyo, Japan). All images were converted to Digital Imaging and Communications in Medicine (DICOM) format (512 × 512 pixels) for analysis, and the CSA of muscle, bone, and fat was measured to the nearest 0.01 cm².

**Figure 1:**
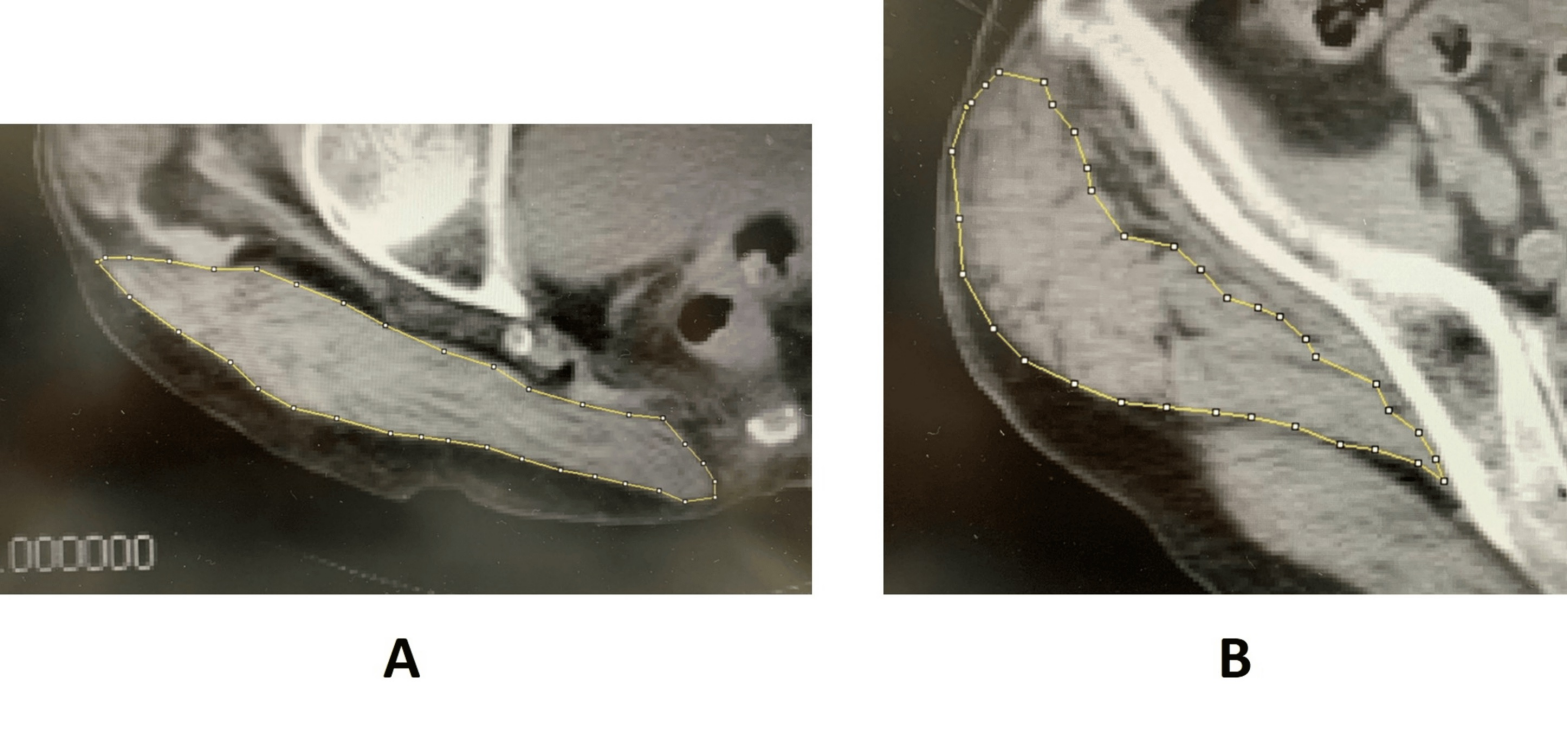
CT images of the gluteus maximus and gluteus medius Axial images at the level of the tip of the greater trochanter represent the gluteus maximus (A), while those at the level of the third sacral vertebra represent the gluteus medius (B).

**Figure 2:**
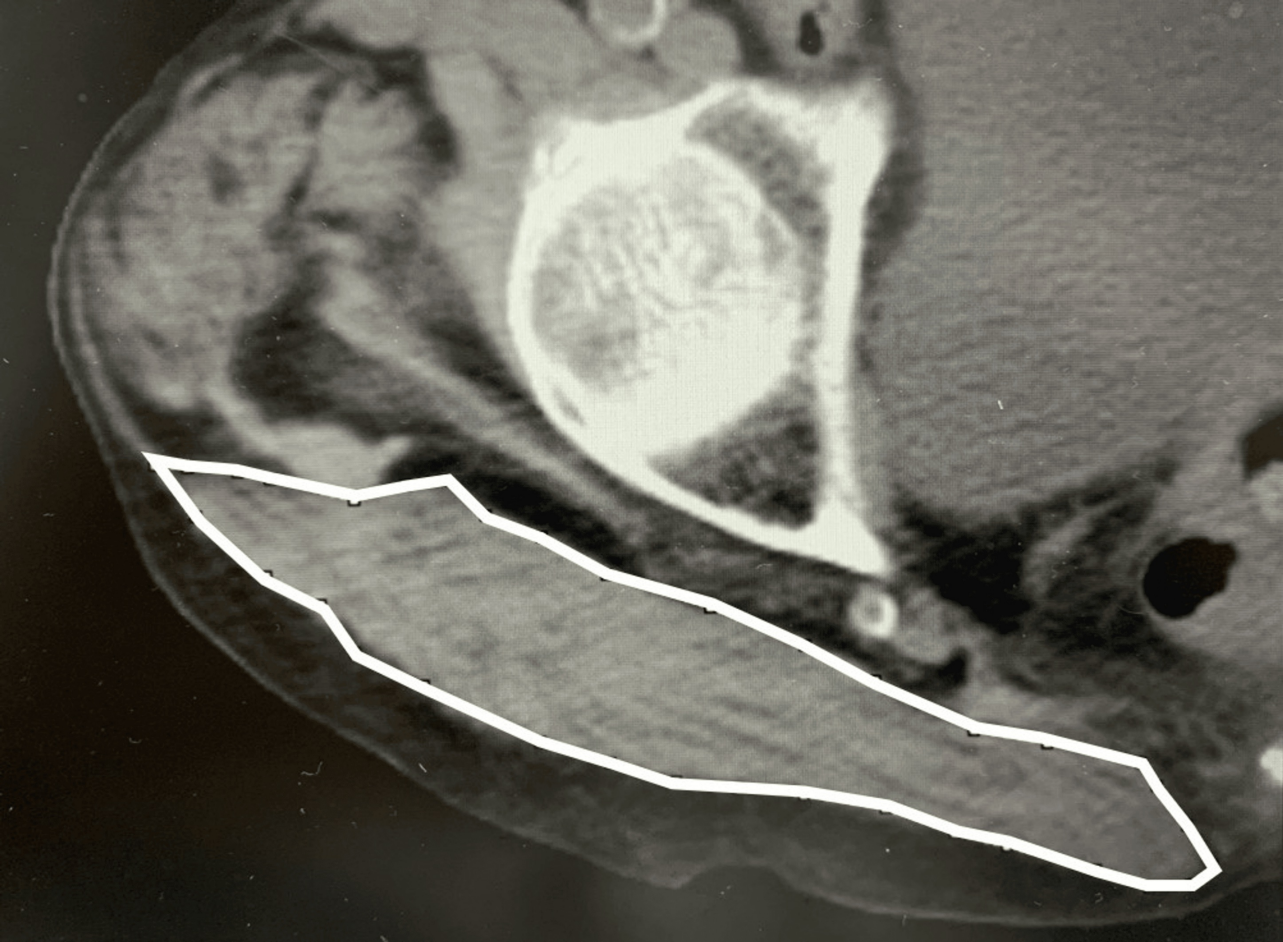
Cross-sectional area of the gluteus medius muscle An observer manually traced the boundaries of the muscle to calculate the cross-sectional area.

In this study, the non-injured side was used to assess muscle data, as fractures on the injured side impaired proper measurement. Axial images were specifically selected as follows: (1) gluteus maximus, at the level of the contralateral greater trochanter tip; and (2) gluteus medius, at the level of the third sacral vertebra (Figures 1A, 1B) [12,13]. The CSA was manually traced and calculated within the contoured area (Figures 2).

Both the CSA and muscle density (measured in Hounsfield Units (HU)) were assessed from the same axial slices for the gluteal muscles. The muscle density was determined by segmenting regions corresponding to adipose tissue (-100 to -50 HU) and higher values associated with muscle tissue. Total HU values were adjusted based on their distribution within the muscle to reflect the overall composition and fat infiltration, as outlined in our preliminary study [14]. A single examiner (M.N.) performed all assessments.

Statistical analysis

Statistical analyses were conducted using EZR (Saitama Medical Center, Jichi Medical University, Saitama, Japan), a graphical user interface for R (The R Foundation for Statistical Computing, Vienna, Austria). A p-value < 0.05 was considered statistically significant.

Univariate analyses of independent variables (age, height, body weight, BMI, fracture laterality, and muscle metrics) were performed. All variables except fracture laterality were treated as continuous and compared between the stable (A1) and unstable (A2) groups using Welch’s t-test or the Mann-Whitney U test, depending on normality. Fracture laterality was assessed using the chi-square test. For multivariate analysis, binary regression was used to estimate odds ratios (ORs) and 95% confidence intervals (CIs) for the risk of unstable fractures associated with covariates derived from muscle and fat indices.

## Results

**Figure 3:**
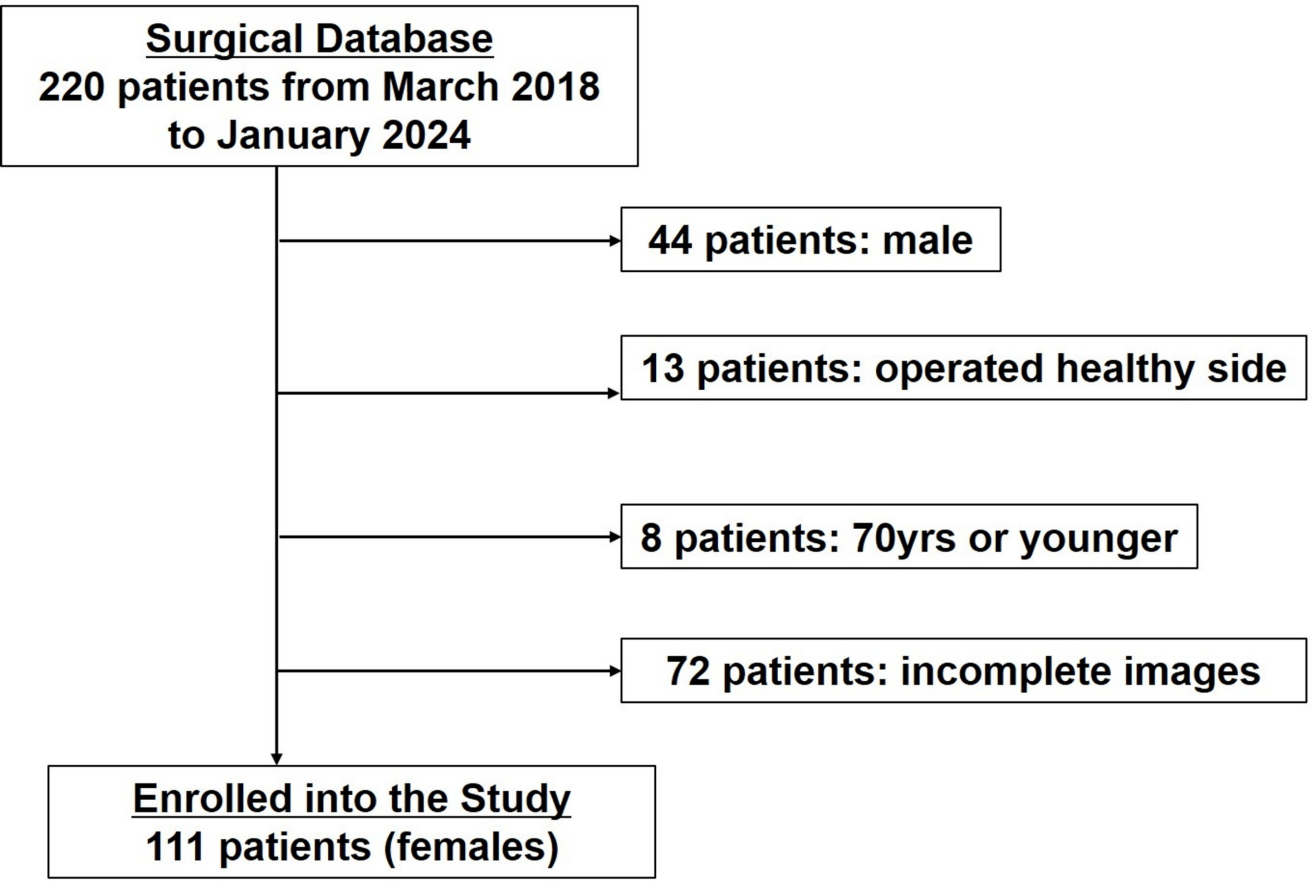
Flowchart of patient selection A flowchart depicting the patient selection process. Some patients were excluded for multiple reasons.

Out of 220 patients identified from the database, 111 patients, with an average age of 88 years (ranging from 71 to 103 years), were included after applying the exclusion criteria (Figure 3). This cohort consisted of 40 patients with stable fractures (A1) and 71 patients with unstable fractures (A2), classified based on the preoperative AO/OTA system (Table 1). Age at surgery, height, and BMI were not significantly associated with fracture stability. However, fracture laterality showed a significant difference between the groups: patients with stable fractures (A1) had a significantly higher proportion of left-sided injuries compared to those with unstable fractures (A2) (p < 0.05). Regarding gluteal muscle data, the CSA/BMI of both the gluteus maximus and medius, as well as the density of the gluteus medius, did not show significant differences between the two groups. The only exception was the density of the gluteus maximus, which was significantly lower in the unstable group (p < 0.01).

**Table 1:** Patient demographics and comparison of variables between stable and unstable groups p-values for group comparisons of each variable were calculated using Welch’s t-test or the Mann-Whitney U test for age, body weight, height, and muscle metrics, and the chi-square test for right vs. left fracture laterality. Continuous variables are presented as mean values with their ranges. * indicates p < 0.05. CSA: cross-sectional area, G.Max: gluteus maximus, G.Med: gluteus medius.

-	Stable (A1) 41 patients	Unstable (A2) 70 patients	p-value
Age (years)	88 (71~103)	88 (75~97)	0.92
Height (cm)	146.6 (129~161)	149 (128~165)	0.25
BMI (kg/m^2^)	19.8 (13.2~33.8)	20.0 (13.2~31.1)	0.18
Left/right	29/12	34/36	0.02*
G.Max CSA/BMI (m²/10^6 ^kg)	116.7 (48.0~179.7)	125.6 (63.7~298.3)	0.18
G.Med CSA/BMI (m²/10^6^ kg)	91.3 (47.2~145.4)	95.7 (40.1~143.7)	0.11
G.Max density (HU)	22.7 (0.2~40.5)	17.2 (-27~41.5)	0.01*
G.Med density (HU)	30.6(-6.9~45.3)	27.3 (-8.5~43.7)	0.14

**Figure 4:**
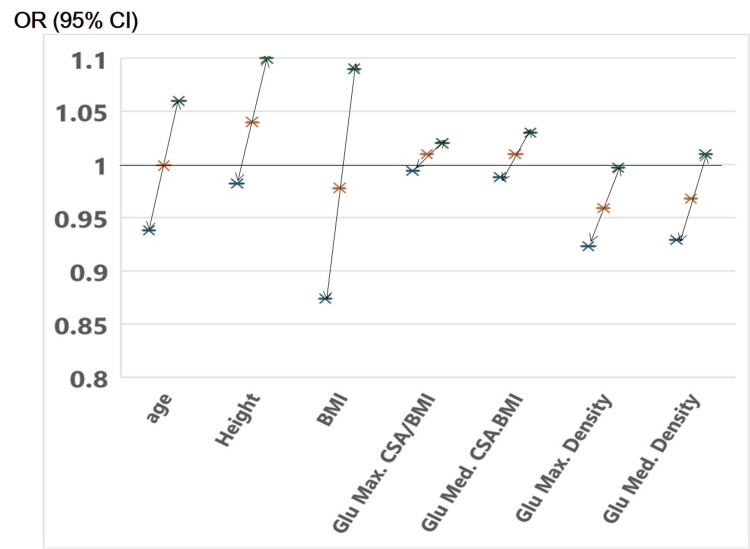
Hazard ratios for unstable fracture risk per one standard deviation decrease This figure illustrates the results of a binary logistic regression analysis assessing factors influencing fracture stability, categorized into "Stable" and "Unstable" groups. Independent variables included age at surgery, height, BMI, and gluteus muscle metrics. Among these variables, only the density of the gluteus maximus was found to be statistically significant in predicting fracture stability. A lower muscle density was associated with an increased likelihood of an unstable fracture. The odds ratio and 95% confidence interval are presented as 0.955 (CI: 0.918-0.994) (p=0.025). CSA: cross-sectional area, Glu Max: gluteus maximus, Glu Med: gluteus medius.

A binary logistic regression analysis was conducted to identify risk factors for unstable fractures. The analysis found that the density of the gluteus maximus was a statistically significant predictor, with an odds ratio of 0.959 (95% confidence interval (CI), 0.923 to 0.997; p = 0.03) (Figure 4).

**Figure 5:**
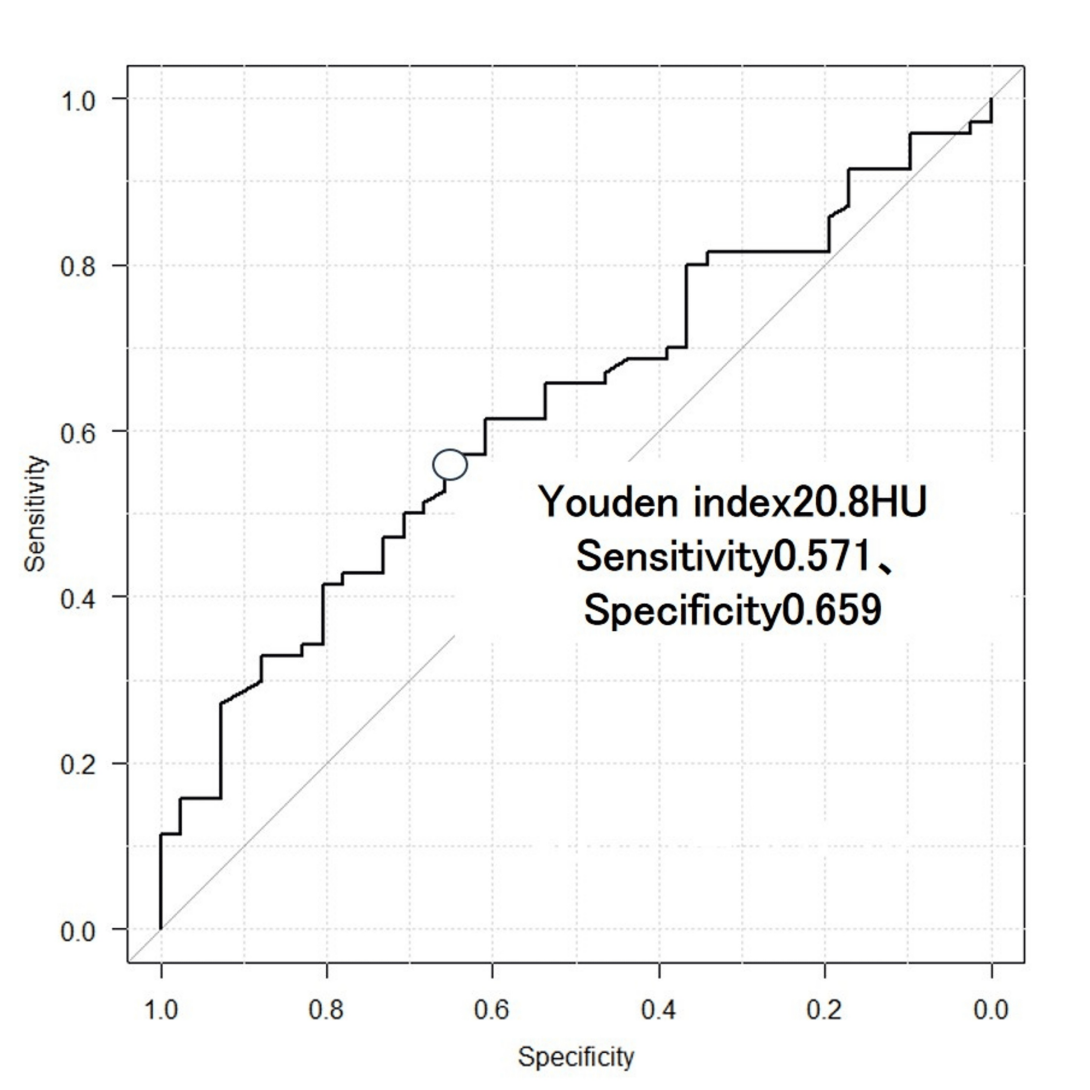
Receiver operating characteristic curve The receiver operating characteristic (ROC) curve illustrates the balance between sensitivity and specificity for the density of the gluteus maximus in distinguishing between stable (A1) and unstable (A2) fractures. The area under the curve (AUC: 0.625; 95% CI: 0.520-0.730) quantifies the accuracy of this metric. The optimal cut-off value, derived using Youden’s index, is 20.8.

To determine an optimal cut-off value, receiver operating characteristic (ROC) analysis was performed for the density of the gluteus maximus (Figure 5). The mean Hounsfield Unit (HU) value (± standard deviation) for the density of the gluteus maximus was 22.7 ± 9.6 HU in the stable group (A1) and 17.2 ± 13.1 HU in the unstable group (A2). The Youden index identified a cut-off value of 20.8 HU for the gluteus maximus density as the optimal threshold (area under the curve (AUC): 0.625; 95% CI: 0.520-0.730).

Case presentation

**Figure 6:**
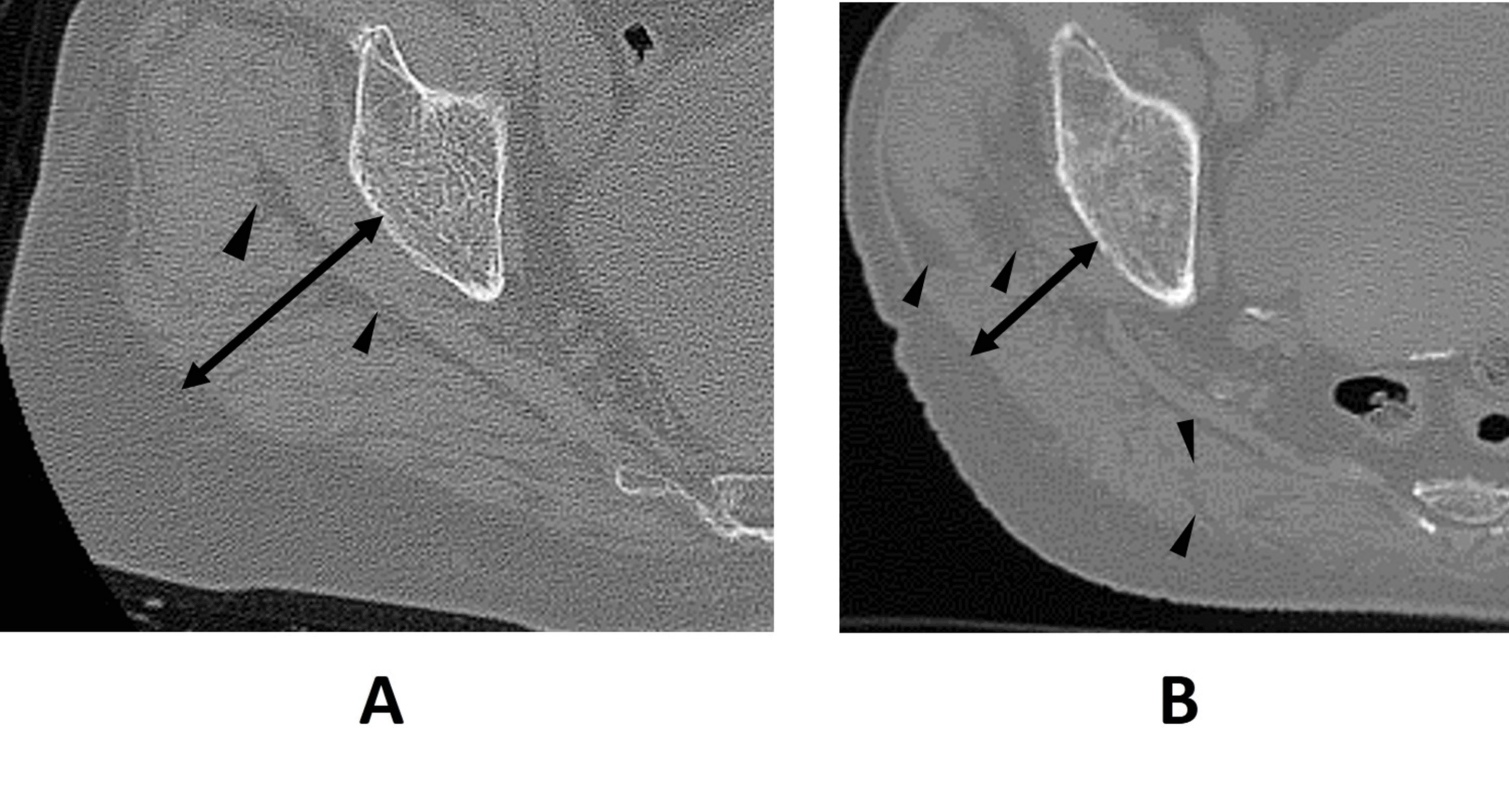
Case presentation Comparison of two axial CT images showing (A) robust muscle with minimal fatty infiltration in a stable fracture and (B) thinner muscle with substantial fat accumulation in an unstable fracture. The image suggests that sufficient muscle mass may help mitigate the impact on the proximal femur. Double-headed arrow: muscle thickness covering the bone; arrowheads: inter- and intra-muscular fat tissue.

Two case studies were presented, illustrating axial CT slices of the non-injured side to contrast gluteal muscle appearance between stable and unstable pertrochanteric fractures. In the case of a stable fracture, the gluteal muscle appeared thick and robust, while the patient with an unstable fracture showed a markedly thinner muscle belly, interspersed with fatty tissue (Figures 6A, 6B).

## Discussion

This study aimed to investigate whether unstable pertrochanteric fractures in elderly patients are associated with weakened gluteal muscles compared to stable fractures. Our binary logistic regression analysis demonstrated a significantly lower density of the gluteus maximus in the unstable fracture group (A2) compared to the stable group (A1), with an odds ratio of 0.959, indicating a notable relationship between muscle weakness and fracture instability. ROC analysis for the gluteus maximus density identified 20.8 as the optimal cut-off value (AUC: 0.625; 95% CI: 0.520-0.730). To our knowledge, few studies have explored the role of muscle quality in determining fracture stability or fragment comminution [13].

The role of soft tissue as a cushion

Soft tissue protection is widely believed to reduce fracture stress, potentially mitigating the extent of fracture fragmentation and leading to more stable fracture patterns [9]. Biomechanical studies using cadavers or finite element models simulating falls have highlighted the importance of soft tissue in absorbing impact energy, identifying this as a critical factor in fracture prevention [9,15]. Each standard deviation decrease in trochanteric soft tissue thickness has been associated with a 1.8-fold increased risk of hip fracture, even after adjusting for soft tissue's force-attenuating capacity [16]. However, Kim et al. [13] noted the dual effects of fat and muscle on bone forces during a fall. While muscle and fat contribute to energy absorption, excess fat can impede a patient’s ability to perform swift protective movements during a fall.

The distinct roles of gluteus maximus and medius as buffers

From an anatomical perspective, the gluteus maximus seems to play a more substantial role in buffering stress at the hip compared to the gluteus medius. The gluteus maximus attaches distally, covering the posterior part of the hip joint, while the gluteus medius inserts near the tip of the greater trochanter without significantly bridging the fracture site [17,18]. In terms of size, the gluteus maximus weighs 573.4 g, significantly larger than the gluteus medius at 251.7 g, and also has over three times the cross-sectional area [19]. These anatomical features align with our findings that the density of the gluteus maximus, not the gluteus medius, correlates with fracture stability.

Other factors contributing to fracture instability

Several factors are known to influence fracture instability. Surveillance studies have demonstrated that elderly and female patients show a statistically significant association with unstable fractures, a finding consistent with our preliminary results [20]. However, other studies dispute this, arguing that the stability of intertrochanteric fractures cannot be predicted based on age, gender, body weight, height, or BMI [9]. Bone mineral density (BMD) is also believed to play a role in fracture stability [9], although present study did not include BMD data. The Revised AO/OTA Classification standardizes lateral wall thickness as a critical metric for postoperative stability in trochanteric fractures, but recent literature has questioned the validity of this theory [21]. Further research is needed to establish a gold standard for fracture stability predictors.

Do unstable fractures lead to poor outcomes?

Unstable fractures have long been associated with poorer outcomes, including higher rates of mortality, medical complications, readmissions, and comorbidities [3,22]. However, questions remain regarding the relative contributions of bone versus muscle factors in these poor postoperative outcomes.

From an osseous perspective, most unstable fractures heal without major complications, largely due to advancements in surgical materials. As long as postoperative shortening remains under 8 mm, the impact on gait performance is minimal. However, when shortening exceeds this threshold, it significantly affects gait, despite showing no difference in functional scores such as the Harris Hip Score and SF-36 Physical Component Score [23,24]. In contrast, muscle fragility is independently linked to increased mortality, complications, and readmissions. A study involving 459 patients, with a median follow-up of 4.5 years, demonstrated that gluteal muscle size and density are significant predictors of mortality in older hip fracture patients, independent of age and clinical risk scores [25]. Furthermore, the cross-sectional areas of the gluteus maximus and medius/minimus on pelvic CT scans are valuable prognostic markers for survival and ICU admission in older patients with proximal femur fractures [26]. In summary, muscle deterioration accompanying unstable fractures likely contributes to poor prognoses.

Limitations

This retrospective study has several limitations. First, we used data from the healthy gluteal muscles, as fractured-side muscles were damaged and unsuitable for measurement. Second, only one examiner conducted the CSA and density measurements. However, the simplicity of the procedure, manually tracing muscle borders, followed by automated calculations, minimized the risk of measurement error, as shown in our preliminary study [14]. Third, muscle metrics were assessed using a single CT slice, without a more comprehensive evaluation of the entire muscular structure [18]. Future studies should assess 3D muscle volume to better understand its cushioning effect. Fourth, certain data that may influence on muscular pathology including comorbidities, medications, serum chemistry, and BMD were not collected for this study. Finally, the sample size was limited.

Strengths

This study has several strengths. First, male patients were excluded to enhance the consistency of muscle-related metrics, as there are significant gender differences in muscle characteristics [27]. Second, only patients aged 70 and older were included, reducing age-related bias. Most patients in the current study were in their 80s or older, reflecting the aging population in our country, and this chronological uniformity increases the study’s reliability. Third, this study is one of the first to focus on the importance of muscle quality in determining fracture stability. Kim et al. [13] pioneered research showing significant differences in BMI-adjusted gluteus maximus and total gluteus area between different stability groups, though without evaluating muscle density.

Innovative insights

This study suggests that poor functional outcomes and higher mortality rates in unstable fractures may be partially due to muscle fragility. Surgeons’ efforts may not fully compensate for reduced function when muscle weakness, a hallmark of aging, is the primary factor [2]. Conversely, some surgeons will advocate for muscle strengthening in elderly patients as a strategy to prevent unstable fractures.

## Conclusions

This retrospective study investigated whether unstable pertrochanteric fractures in elderly female patients were linked to weakened gluteal muscles, compared to stable fractures, suggesting that this muscle weakness may contribute to poor functional outcomes. Our binary regression analysis indicated that decreased muscle density in the gluteus maximus increases the risk of unstable fractures. This result may open new avenues for research into the interplay between muscle pathology and fracture stability.
